# Supplementary material for: Drought and UV Radiation Stress Tolerance in Rice Is Improved by Overaccumulation of Non-Enzymatic Antioxidant Flavonoids
Source: Antioxidants (Basel). 2022 May 6;11(5):917. doi: 10.3390/antiox11050917 (PMC9137601; doi:10.3390/antiox11050917)
Supplement: Supplementary file 1 [file antioxidants-11-00917-s001.zip › antioxidants-1699399-supplementary.pdf]

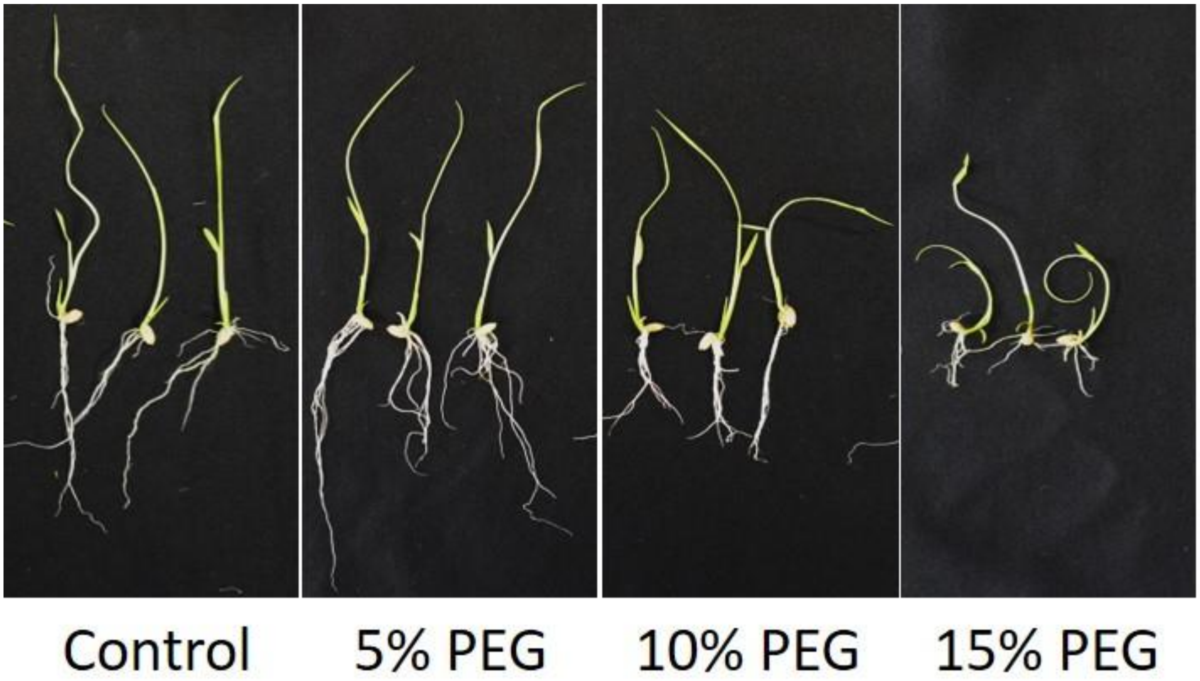

Supplementary Figure S1. Screening of rice seedling on different concentration of PEG 6000.

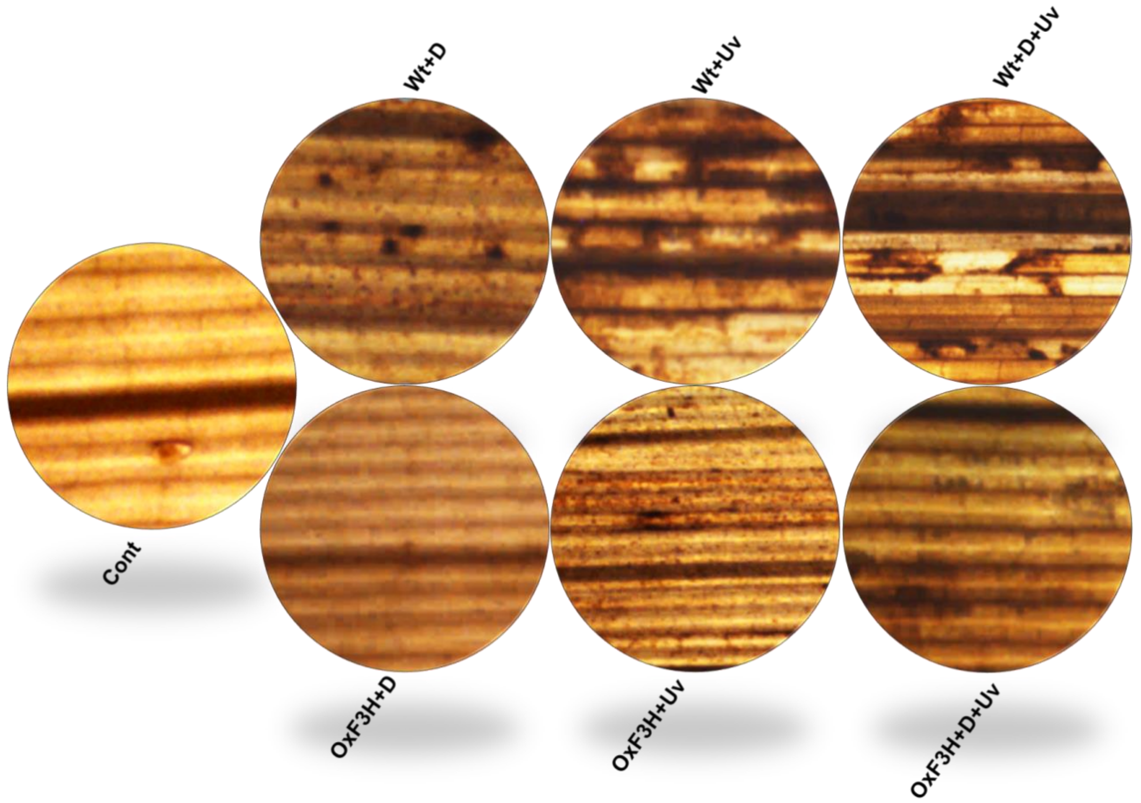

Supplementary Figure S2. Close image of detection of oxidative stress damage, stained with DAB.

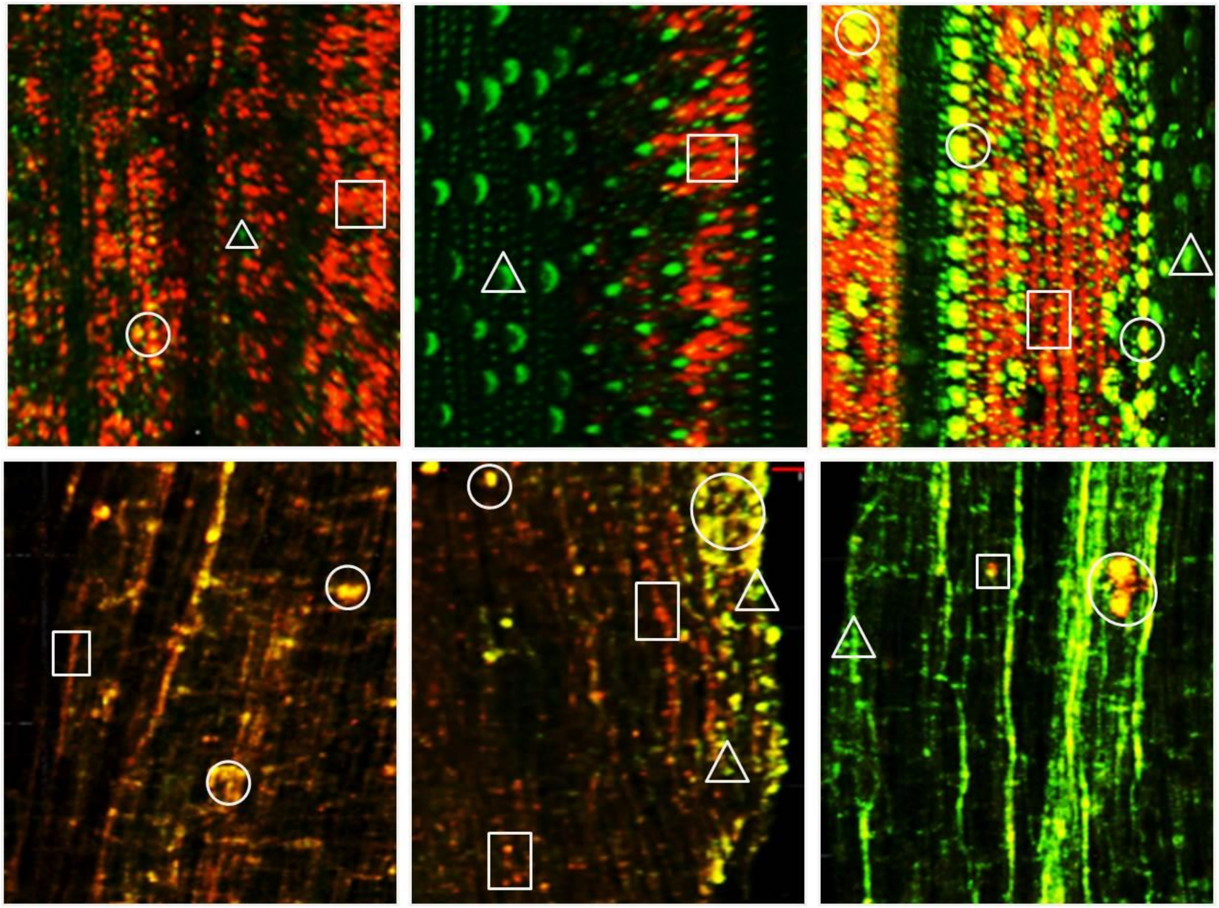

Supplementary Figure S3. Close image of detection of kaempferol, quercetin and naringenin. **Δ** indicates kaempferol, **O** indicates quercetin and □ indicates naringenin.

Supplementary Table S1: List of genes, primers and accession numbers

S.No	Gene name	Primer sequence	Accession number
1	F3H	ATGGCGCCGGTGGCCACGAC (sense)<br>GATGAATCCGCCCTTCTTGCCG (antisense)	MT980835.1
2	UVR8	AGCGGGTGAGGATTTCTTGG (sense)<br>GCTAATCGCCCCGATTCTGA (antisense)	XM_015764478
3	DHN	GTCTCCGTGGAAGAGCCAAA (sense)<br>GTAACCGGGCAGTTTCTCCA (antisense)	XM_015771723

Supplementary Table S2: HPLC conditions used for analysis and quantification of the SA

Equipment	Shimadzu LC-10
Column	HP hypersil ODS (particle size 5μm, pore size 120Å)
Wavelength	Excitation 305nm, Emission 365nm
Detector	RF-10Axl (fluorescence detector)
Solvent A	100% MeOH
Solvent B	100% water in 0.5% acetic acid
Flow rate	1.0mL/mint
